# Genetic and ecophysiological evidence that hybridization facilitated lineage diversification in yellow *Camellia* (Theaceae) species: a case study of natural hybridization between *C. micrantha* and *C. flavida*

**DOI:** 10.1186/s12870-023-04164-4

**Published:** 2023-03-22

**Authors:** Sujuan Wei, Qiwei Zhang, Shaoqing Tang, Wenbo Liao

**Affiliations:** 1 State Key Laboratory of Biocontrol and Guangdong Provincial Key Laboratory of Plant Resources, School of Life Sciences, Sun Yat-Sen University, Guangzhou, 510275 China; 2 Key Laboratory of Ecology of Rare and Endangered Species and Environmental Protection, Ministry of Education, Guangxi Normal University, Guilin, 541004 China

**Keywords:** Yellow camellias, Limestone karst, Hybridization, Narrow endemic, ddRAD, Drought tolerance

## Abstract

**Background:**

Hybridization is generally considered an important creative evolutionary force, yet this evolutionary process is still poorly characterized in karst plants. In this study, we focus on natural hybridization in yellow *Camellia* species, a group of habitat specialists confined to karst/non-karst habitats in southwestern China.

**Results:**

Based on population genome data obtain from double digest restriction-site associated DNA (ddRAD) sequencing, we found evidence for natural hybridization and introgression between *C. micrantha* and *C. flavida*, and specifically confirmed their hybrid population, *C*. “*ptilosperma*”. Ecophysiological results suggested that extreme hydraulic traits were fixed in *C*. “*ptilosperma*”, these being consistent with its distinct ecological niche, which lies outside its parental ranges.

**Conclusion:**

The identified hybridization event is expected to have played a role in generating novel variation during, in which the hybrid population displays different phenological characteristics and novel ecophysiological traits associated with the colonization of a new niche in limestone karst.

**Supplementary Information:**

The online version contains supplementary material available at 10.1186/s12870-023-04164-4.

## Background

Natural hybridization is known to occur in numerous lineages, leading to diverse possible evolutionary consequences [1–3]. In many case, hybridization simply result in the formation of sterile or maladapted offspring [4, 5]. In other case, hybridization may have contributed to speciation and adaptation by generating genetic variation, functional novelty and even new species [3, 6, 7]. Hybridization occurs more frequently among closely related species and is pervasive in recently diverged lineages, such as those that underwent rapid species radiation [8–10]. Such radiation is most likely to happen in mountainous regions, islands and rift-lakes, where alternative resources are more widely accessible [8, 11]. In the context of adaptive radiation, the first step after contact between two established species is the ensuing formation of a hybrid swarm with novel phenotype, which may enable its colonization of novel niches [6, 12]. If hybrids can colonize such niches, become stabilized and eventually lead to speciation [4, 12, 13]. Introgression is also a frequent outcome of hybridization [14]. Adaptive introgression occurs when the transfer genetic material has positive fitness consequences in the recipient species, and is observed between closely related species during the adaptation to new environments [15, 16]. Ecological isolation limits or prevents gene flow and is of itself an important reproductive barrier [17, 18]. Further, the emergence of a new genotype may be directly associated with reproductive isolation, as is the case stark phenological differences arise [19, 20]. Hybridization has now emerged as an important source of adaptation variation, which is quickly introduces much more genetic variation than de novo mutation, and often fuels rapid diversification [2, 15, 21].

Karst landforms are edaphically (soil-related) special terrestrial habitats whose floral composition is unique [22]. In southwestern China, karst terrain with sharp peaks, steep slopes, and deep valleys are conspicuous landscape features, being separated from other outcrops by lowland areas composed of differing soil types [23]. These habitats support high levels of species richness and endemism [24]. Numerous studies have provided valuable insights into karst ecosystems from ecological, physiological, and genetic perspectives, and have greatly improved our understanding of the mechanisms underpinning current species diversity in southwestern China’s karst region. In short, these findings demonstrate that karst forests are influenced by edaphic and hydrological factors related to highly heterogeneous topographies [25, 26]. That body of studies has also revealed that functional trait variation of karst plants influences their species distribution and coexistence [23, 27, 28], and that geographic isolation, genetic drift and selection promotes population differentiation [29–33]. These studies have also reported on the occurrence of whole genome duplication (WGD) events that probably contributed to plants’ adaptation in limestone karst habitats [34]. It is known that hybridization is common in species-rich genera (*Begonia*) in karst regions [35–37]. Karst landforms harbor remarkably high level of plant diversity and given that hybridization is widely acknowledged as a creative force in plant evolution [38], but no study has yet examined hybridization as an aspect of evolution in the diversification of karst plants.

Yellow *Camellia* species (simply referred to as ‘yellow camellias’), are a group of *Camelllia* plants whose flowers are yellow. According to the *Flora Reipublicae Popularis Sinicae* along with subsequent reports of newly described species, there are more than 20 species of yellow camellias that are confined to small areas of southwestern China [39–44]. Yellow camellias have very high morphological diversity, including differences in floral color and structure, and leaf morphology. They also display pronounced habitat preferences, with most species associated with limestone substrates and only four species found growing in acidic soils. Only a single known species occurs naturally in both karst and non-karst soils. Such stark differentiation occurs across a range of topographical positions. For example, on a single mountain, *C. flavida* occupies its karst valley while *C. perpatua* is restricted to its upper slope near to peak. In addition, several interspecific hybridization events have been inferred among the yellow *Camellia* plants growing in China [45]. The high richness of species in close proximity to each other, their high degree of habitat specialization, in addition several suspected reticular events together make yellow camellias an excellent model for studying the potential role of hybridization in diversification events in karst regions.

**Fig. 1:**
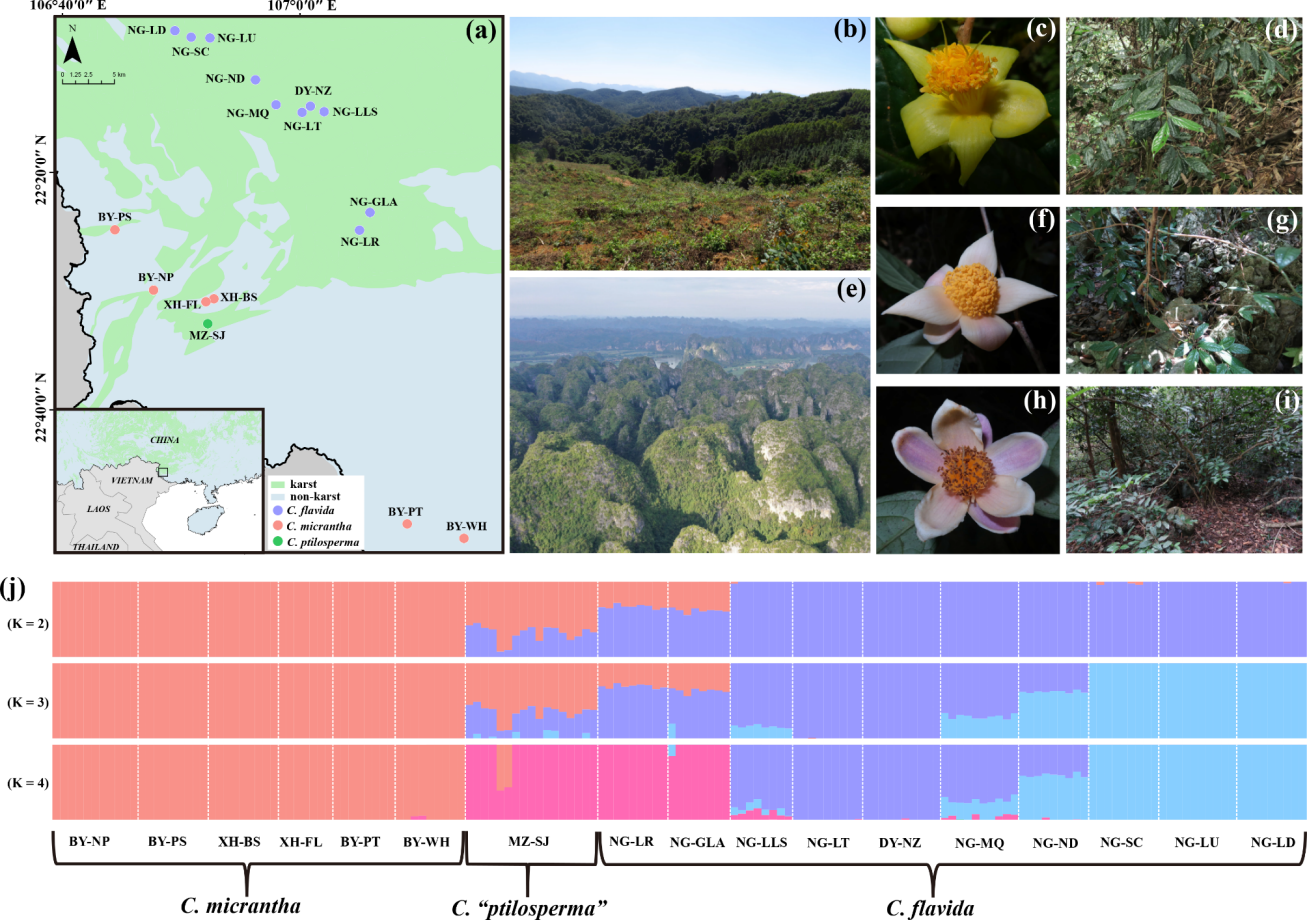
Geographic distribution, morphology, and habitats of the three yellow *Camellia* taxa studied herein. (**a**) Sampling locations in southwestern Guangxi, China. (**b**) Non-karst landscape. (**c-d**) Morphology and habitat of *C. micrantha*. (**e**) Karst landscape. (**f-g**) Morphology and habitat of *C.* “*ptilosperma*”. (**h-i**) Morphology and habitat of *C. flavida*. (**j**) Population structure of the three yellow *Camellia* taxa based on analyses with the ADMIXTURE program at K = 2, 3, and 4

Both *C. micrantha* and *C. flavida* are morphologically distinguishable and favor different habitats: *C. micrantha* grows exclusively in non-karst forests (Figs. 1a, 1b, 1c and 1d), while *C. flavida* typically inhabits karst depressions (Figs. 1a, 1e, 1h and 1i). Karst soils are typically shallow and are characterized by lower water storage capacity in comparison to the surrounding non-karst soils [46]. Therefore, karst plants frequently incur drought because of low soil water availability, particularly in the dry season [47, 48]. Karst and non-karst species also differ in their hydraulic traits in correspondence with their distribution patterns [28]. Despite this, a plant known as *Camellia ptilosperma* is suspected to have originated via hybridization between *C. micrantha* and *C. flavida* based on the inconsistencies in the phylogenies derived from nuclear and chloroplast genomes [45]. Morphologically similar to *C*. *flavida* (Fig. 1f), *C*. *ptilosperma* is primarily characterized by long flowering period that distinguish it from other yellow *Camellia* species, hence why it was initially reported as being a new species [49], albeit later merged with *C. flavida* [50]. The distribution of this putative hybrid (herein *C.* “*ptilosperma*”) is restricted to a very limited area (less than 50 km^2^, Fig. 1a), in geographic proximity of *C. micrantha*. It grows on karst slopes, across elevation gradients spanning 230 to 390 m above sea level, characterized by extensive rock outcrops and shallow soils (Fig. 1g). In karst, the soil depth as well as soil distribution continuity generally decrease as the elevation increases [51, 52]. Water availability is the key factor determining the distribution of karst plant species [25, 27], that of *C.* “*ptilosperma*” implies these plants probably be more drought-tolerant than species growing in karst valleys/depressions and where the slopes are low. It is speculated that a novel ecological preference might have emerged in *C*. “*ptilosperma*”. In theory, tolerance of such habitat in a hybrid population could be conferred by fixation of extreme traits [6, 53, 54]. However, the hybrid origin of *C.* “*ptilosperma*” remains untested at population genetic level, and whether *C.* “*ptilosperma*” has accordingly achieved niche divergence has not been determined, leaving us with nothing known about its drought tolerance ability.

To this end, here we employed double digest restriction site-associated DNA sequencing (ddRAD-seq) for genomic analysis, and investigated the ecophysiological features associated with habitat adaptation. The specific aims of this study were to: (1) test leading hypotheses about the hybrid origin of *C.* “*ptilosperma*”, and (2) to explore whether *C.* “*ptilosperma*” has a greater tolerance of specific habitat stresses than its parent species. The obtained data and findings provide for a possible mechanistic basis for lineage diversification in yellow camellias.

## Results

### SNP genotyping

A total of 2 441 209 808 (mean ± standard deviation [SD], 46 060 562 ± 22 329 937 per individual), 4 004 099 068 (44 001 088 ± 16 071 811 per individual), and 582 841 776 (34 284 810 ± 9 872 024 per individual) reads were obtained from the 53, 91, and 17 individuals of *C. micrantha*, *C. flavida*, and *C.* “*ptilosperma*”, respectively. The dataset obtained using the STACKS program contained 705 554 SNPs. After filtering, the 4422 SNPs obtained and used for subsequent analyses.

### Population structure

Analyses of genetic variations from genome-wide SNP data using the cross-validation (CV) method in ADMIXTURE revealed an optimal value of K = 4 (Fig. S1). For K = 2, the *C.* “*ptilosperma*” population showed a high degree of admixed ancestry, with ca. 68% was derived from *C. micrantha* and ca. 32% derived from *C. flavida*. Moreover, *C. flavida* further diverged into different lineages at K = 3 (Fig. 1j). The NG-LLS, NG-MQ, and NG-ND populations showed genetic admixture (Fig. 1j) and the remaining populations showed 100% pure assignment to their respective clusters. For K = 4, most individuals of MZ-SJ population and the *C. flavida* populations NG-LR and NG-GLA formed a single genetic cluster. Two individuals of the MZ-SJ population still showed a high degree of admixed ancestry with *C. micrantha* where K = 4 (61% and 57%, Fig. 1j). *C. micrantha* remained genetically homogeneous across all levels of partitioning, except for the BY-WH population, which featured limited admixture with a cluster comprising the MZ-SJ, NG-LR, and NG-LT populations at K = 4 (Fig. 1j). The ADMIXTURE results also revealed that the NG-LR and NG-GLA populations of *C. flavida* harbored some degree of introgression from *C. micrantha* (33%), this being especially distinct at K = 2 and K = 3 (Fig. 1j).

**Fig. 2:**
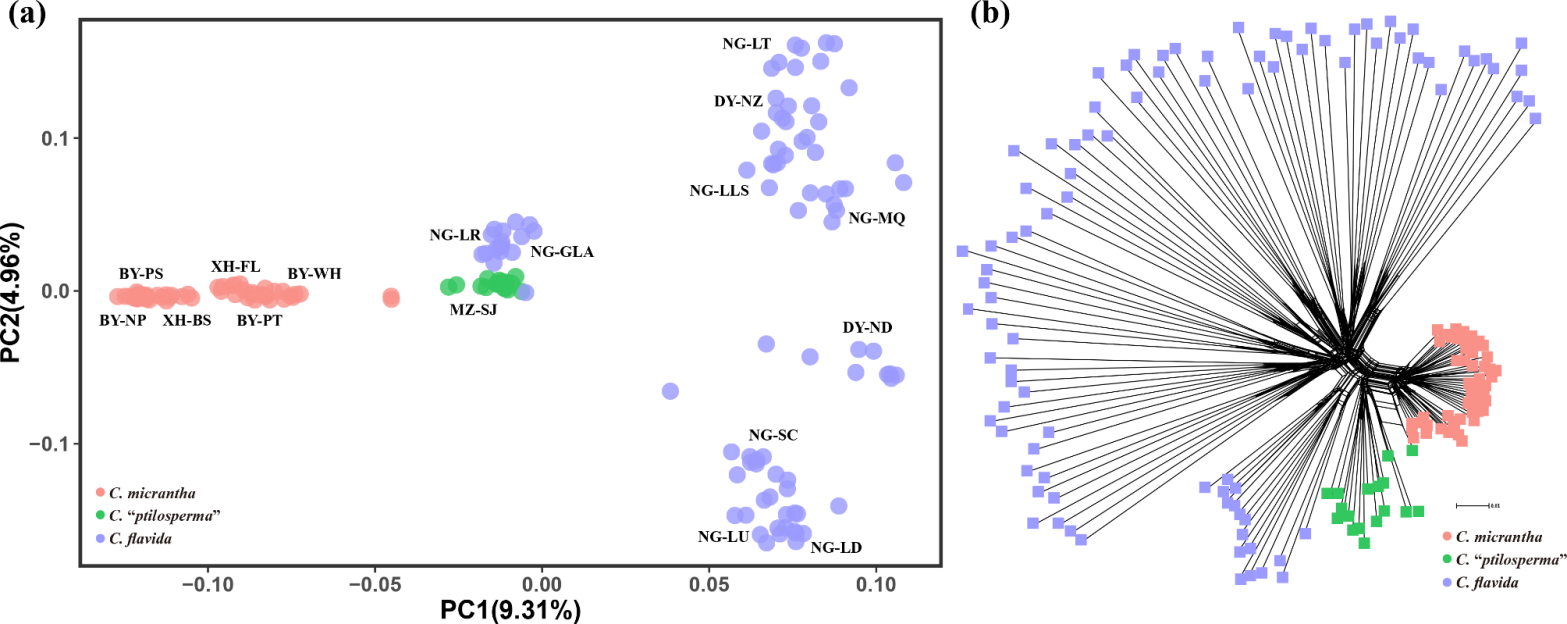
Genetic analyses of *C. micrantha*, *C.* “*ptilosperma*” and *C. flavida*. (**a**) Principal component analysis (PCA) plot of the first two components. (**b**) Phylogenetic network showing genome reticulation. Branch lengths are proportional to absolute distances calculated from the binary matrix

The principal component analysis (PCA) results uncovered four major clusters, which corroborated the result obtained from the ADMIXTURE analysis (Fig. 2a). In this respect, *C. micrantha* and *C. flavida* were well differentiated from each other along PC1, which explained 9.31% of the variance. The individuals of *C.* “*ptilosperma*” and the NG-LR and NG-GLA populations of *C. flavida* that showed genetic introgression had intermediate values along PC1, yet were distinguishable along PC2, which explained 4.96% of the variance (Fig. 2a). The constructed neighbor-network based on the SNP data was also consistent with the genetic pattern evident from ADMIXTURE analyses and the PCA biplot. All the individuals of *C. micrantha* formed a single group that was positioned close to the group formed by individuals of *C.* “*ptilosperma*” (Fig. 2b). All *C.* “*ptilosperma*” individuals clustered at the intersection of the splits between *C. micrantha* and *C. flavida* (Fig. 2b).

### Genetic diversity and differentiation statistics

**Fig. 3:**
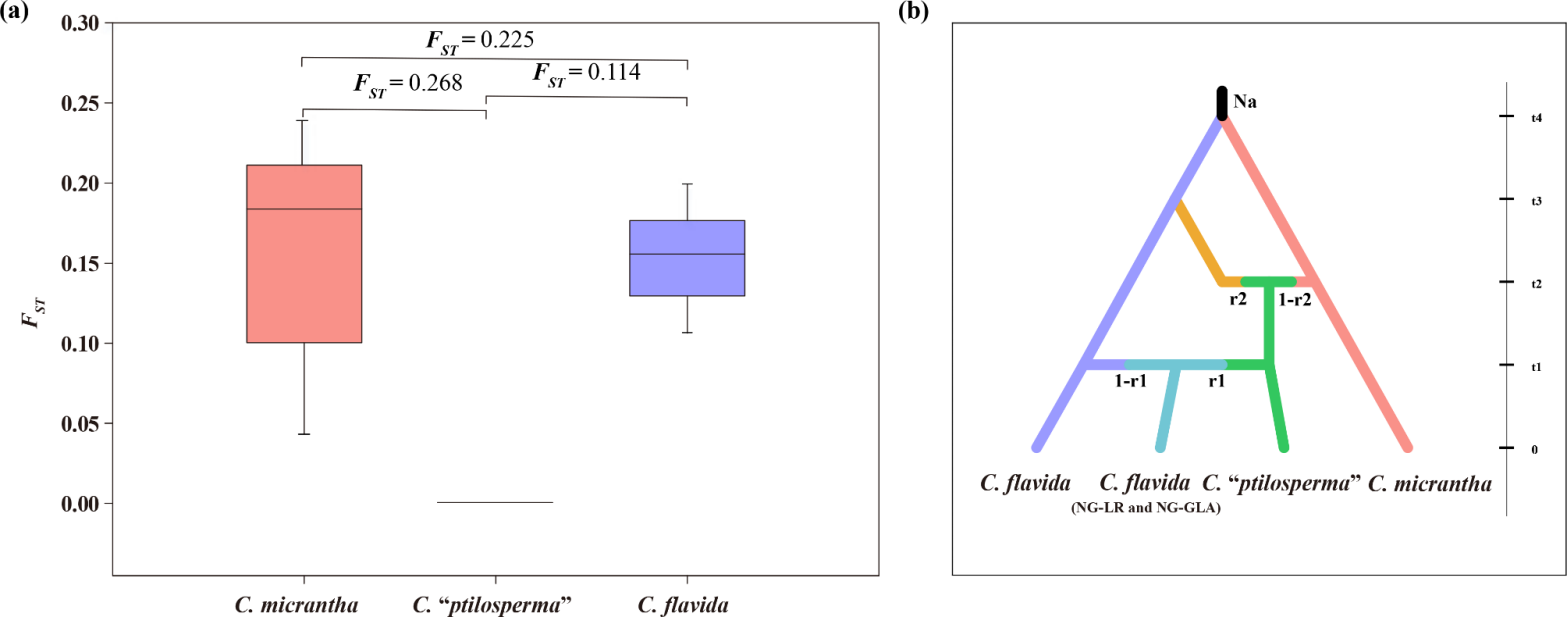
Genetic differentiation of *C. micrantha*, *C.* “*ptilosperma*” and *C. flavida* and the population history scenario examined in DIYABC. (**a**) *F*_*ST*_ within each taxa and between pairs of taxa based on the obtained genome-wide SNP data. (**b**) Scenario 1 was selected as being the most likely describe the origin of admixed lineages. Generations are shown on the y-axis (t0 to t4) and admixture proportions from each parent are shown on the scenario

The genetic diversity of *C. flavida* was estimated to be higher than that of either *C.* “*ptilosperma*” or *C. micrantha* (Table S1). All the *C. flavida* populations harbored extremely high within-population genetic variation, as reflected in all the measures of genetic variation estimated here; in stark contrast, genetic diversity was extremely low for all populations of *C. micrantha* (Table S1). The estimated *F*_*ST*_ values indicated a slightly low differentiation between *C.* “*ptilosperma*” and *C. flavida* (*F*_*ST*_ = 0.114). The genetic differentiation was greater between *C.* “*ptilosperma*” and *C. micrantha* (*F*_*ST*_ = 0.268) than between *C. micrantha* and *C. flavida* (*F*_*ST*_ = 0. 225) (Fig. 3a).

### ABC-based inferences of population history

In total, 161 individuals from 17 populations were examined. DIYABC analysis is based on a total of 415 SNPs after exclusion of monomorphic loci. The posterior probabilities for scenario 1 was 0.6317 (95% confidence interval (CI) 0.6097–0.6536), much higher than for the other nine scenarios (Fig. S2). For scenario 1, the median values of the effective population sizes of N1 (*C. micrantha*), N2 (*C.* “*ptilosperma*”), N3 (NG-LR and NG-GLA populations of *C. flavida*), N4 (*C. flavida*) and Na were 7.73 × 10^3^, 3.30 × 10^3^, 1.06 × 10^3^, 1.25 × 10^4^, 8.20 × 10^3^, respectively (Fig. S3). The median values of the divergence time, t1, and the time of hybridization event, t2, were 97.9 (95% CI: 16.5–275) and 211 (95% CI: 42.8–1340) generations ago, respectively (Fig. S3). Principal component analyses showed that the summary statistics of observed datasets was similar to simulated datasets (Fig. S4), suggesting that scenario 1 was generally fitted to the observed data.

Results of approximate bayesian computation (ABC) approach showed that the most supported scenario (Scenario 1) was the one considering *C.* “*ptilosperma*” arose hybridization events between *C. micrantha* and *C. flavida*, and populations of NG-LR and NG-GLA was originated from introgression of *C. micrantha* genes into *C. flavida* (Fig. 3b).

### Variation in pressure-volume (P-V) traits

**Fig. 4:**
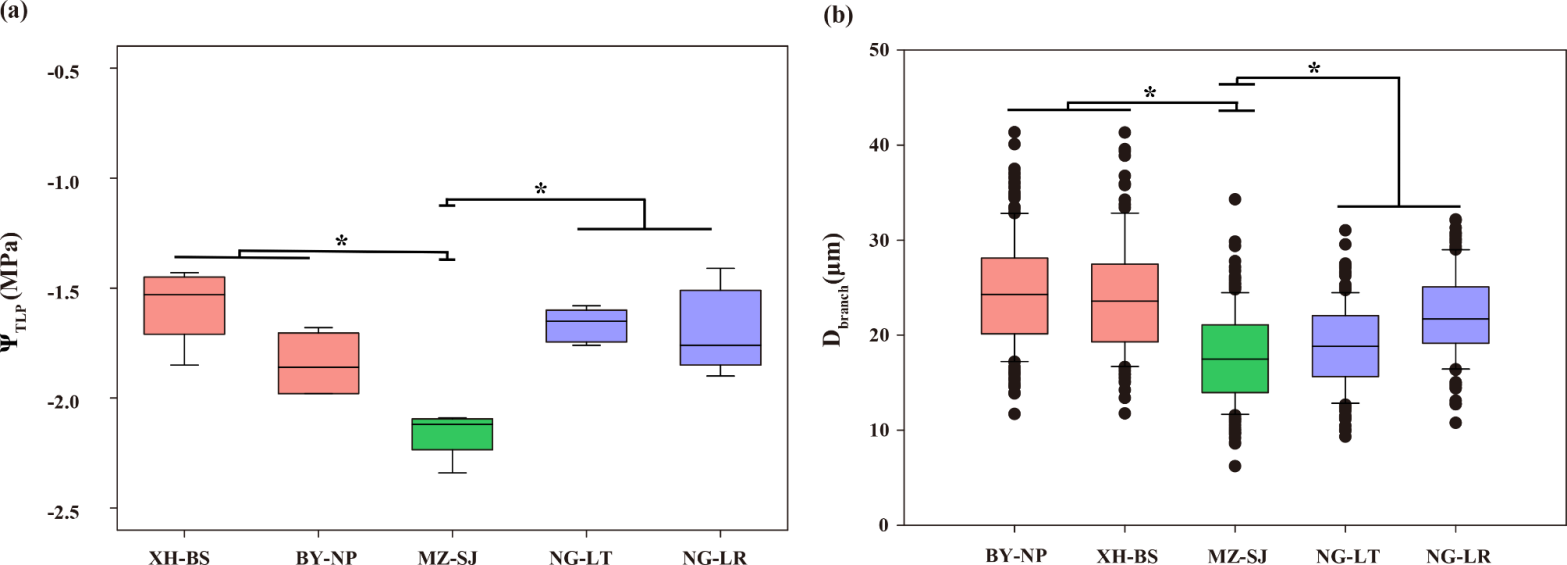
The value of Ψ at turgor loss point (Ψ_TLP_) and diameter of the vessels in the branches. (**a**) Comparison of the significantly negative value of Ψ_TLP_ of *C.* “*ptilosperma*” with the Ψ_TLP_ values of *C. micrantha* and *C. flavida*. * p < 0.05. (**b**) The diameter of the vessels in the branches of *C.* “*ptilosperma*” was significantly different from those of *C. micrantha* and *C. flavida*. * p < 0.05

Of all the populations of yellow camellias studied here, significant differences were observed in the leaf P-V parameters of *C.* “*ptilosperma*” vis-à-vis its putative parental species (Fig. 4a). The value of Ψ at turgor loss point (Ψ_TLP_) for the *C.* “*ptilosperma*” was significantly more negative than that of either XH-NP (p = 0.01810) and XH-FL (p = 0.00002) populations of *C. micrantha* and likewise for the NG-LT (p = 0.00020) and NG-LR (p = 0.00041) populations of *C. flavida*. No significant differences were observed between the Ψ_TLP_ of *C. micrantha* and *C. flavida* (Fig. 4a).

### Capacitance and wood properties

The water content of different *Camellia* taxa and tissues is presented in Table S2 and the corresponding water release curves of the branches and roots of the yellow *Camellia* species are depicted in Fig. S5. Branch or root capacitance did not differ significantly among the taxa studied (Table S2, Fig. S5). These results indicated that the yellow *Camellia* taxa we examined all have similar water storage capacity.

Nevertheless, the vessel diameters were narrowest in the branches of *C.* “*ptilosperma*”, and there were significant differences between the vessel diameters of *C.* “*ptilosperma*” in comparison with its putative parental species (Fig. 4b). Aside from that, however, there were negligible differences among these yellow *Camellia* taxa in their wood characteristics, including fibers/tracheids (F), and ray and axial parenchyma (RP and AP) (Table S3).

## Discussion

### Hybridization occurs between ***C. micrantha*** and ***C. flavida***, resulting in ***C.*** “***ptilosperma***”

This study’s results supported naturally occurring hybridization and introgression between *C. micrantha* and *C. flavida* in narrow sympatric zone of southwest Guangxi (China), despite their strong morphological and ecological differentiation. The evidence for this came from a suite of complementary results for gene clustering, PCA, and the phylogenetic network analysis based on SNP data (Figs. 1 and 2).

In the ADMIXTURE analyses, population MZ-SJ of *C.* “*ptilosperma*” and the populations NG-LR and NG-GLA of *C. flavida* present a different degree of admixed ancestry and appear to constitute an independent cluster when K = 4 (Fig. 1j). However, a recent phylogenomics study revealed a conflicting relationship for *C.* “*ptilosperma*”, with it found more closely related to *C. micrantha* in the chloroplast phylogeny, whereas it was genetically closer to *C. flavida* in the phylogenetic trees constructed with nuclear DNA [45]. In contrast to *C.* “*ptilosperma*”, individuals from NG-LR and other populations of *C. flavida* show consistent relationships in all phylogenies [45]. Further, earlier it was found that only one chloroplast haplotype was observed in the *C.* “*ptilosperma*” population and it is genetically distinct from all haplotypes of *C. flavida* [55]. Altogether, this indicates that population MZ-SJ of *C.* “*ptilosperma*” and the populations NG-LR and NG-GLA of *C. flavida* have a distinct evolutionary origin. Approximate Bayesian computation result support scenario 1 as being the most likely one to have occurred, which suggests that hybridization events between *C. micrantha* and *C. flavida* gave rise to *C.* “*ptilosperma*”, and backcrossing led to the *C. flavida* populations NG-LR and NG-GLA being composed entirely of advanced of introgression individuals (Fig. 3b). When using K = 2, and thus intermediate to the parents, the ADMIXTURE analyses of both data sets demonstrated that *C.* “*ptilosperma*” showed greater similarity to *C. micrantha* (Fig. 1j). This result agrees with the expectation that the hybrid genome is unlikely to inherit an equal proportion from each parent as backcrossing is expected to occur [4]. Additionally, the different proportion of genetic material for the parental species to the genome of the hybrid could also be explained by the selection against minor parent ancestry [56].

Overall, population MZ-SJ (*C.* “*ptilosperma*”) is likely of hybrid origin and subsequently backcrossing with its parents, suggesting it could be a hybrid swarm. Specifically, *C. micrantha* served as the maternal parent while *C. flavida* served as the paternal parent during the initial hybridization event.

### Novel hydraulic traits in ***C.*** “***ptilosperma***” support its distinct niche divergence

The ecophysiological experiments revealed that the focal taxa exhibited inherent differences in their hydraulic traits related to water availability. In particular, we find that the water potential at turgor loss point (Ψ_TLP_) as well as vessel diameter in branches of *C.* “*ptilosperma*” differed significantly from its parental species.

Specifically, the Ψ_TLP_ values of *C.* “*ptilosperma*” were significantly lower than those of its parents (Fig. 4a). The maintenance of cell turgor in leaves is crucial for plants to avoid injury and/or for maintaining cellular functions during drought conditions [57, 58]. A more negative value of Ψ_TLP_ indicates a greater ability to withstand water deficits [59–61]. The significantly greater negative value of Ψ_TLP_ in *C.* “*ptilosperma*” suggests it has a survival advantage during the colonization of habitats where water availability is limited. We also observed an extremely narrow diameter of vessels in the branches of *C.* “*ptilosperma*” that was highly significantly different from those of its parental species (Fig. 4b). A similar pattern has been reported in other plant species growing in tropical karst forests [28]. This low vessel diameter indicates that *C.* “*ptilosperma*” compromises hydraulic efficiency for hydraulic safety, and this underlying mechanism could ensure survival in arid environments [62, 63].

Transgressive segregation is considered an important mechanism by which hybridization promotes ecological transition [4, 17, 18, 38, 64–69]. When hybrids exhibit novel or extreme characters vis-à-vis parental taxa, it is termed ‘transgressive segregation’, a phenomenon typically observed in early-generation hybrids [6, 54, 68]. For instance, in two alpine hybrid species their fixed novel drought-tolerance traits enable their persistence in the harsh alpine habitat [70, 71]. With the exception of genetic variation, phenotypic plasticity in hydraulic traits is another factor of adaptation [72–74]. Drought-tolerance traits in *C.* “*ptilosperma*” is also likely resulting from phenotypic plasticity. This emphasizes the need for conducting common garden experiments to confirm the origin of novel hydraulic traits in *C.* “*ptilosperma*”.

Our study demonstrated that compared with those of its parental species, *C.* “*ptilosperma*” had extreme ecophysiological traits related to drought tolerance. These findings suggest that *C.* “*ptilosperma*” has acquired novel traits lacking in either parent, and which are consistent with its adaptation to niches that are unfavorable to both its parental species.

### Hybridization as a mechanism driving lineage diversification in yellow camellias

Hybridization is considered a producer of genetic variation that provides species at early stages of differentiation with prerequisite evolutionary novelties [12, 75, 76]. Such novel phenotypes may facilitate adaptive invasion of hybrid organisms into novel ecological niches, which enables ecological differentiation between hybrid progenies and parental species [4]. Additionally, divergent phenology can also decrease gene flow between hybrids and parents, thereby favoring hybrid establishment and subsequent independent evolution [12, 17, 65].

The hybrid derivative *C.* “*ptilosperma*” appears to conform to this expectation. Indeed, our study provides substantial, compelling evidence regarding the hybrid origin of *C.* “*ptilosperma*”. Ecophysiological results also confirm that extreme characters (significantly negative value of Ψ_TLP_ and smaller vessel diameter) have become well fixed in *C.* “*ptilosperma*”, conferring to it a greater ability to withstand drought, thus allowing it to grow on karst hillslopes where they must rely on shallow soil water. Moreover, *C.* “*ptilosperma*” differs in a key phenological trait from its ancestral parents. Best known for its long flowering period (from May to December, with the peak of flowering in July) [49], *C.* “*ptilosperma*” had been considered as the candidate for generating new cultivated varieties in yellow-flower *Camellia* breeding [77, 78]. Interestingly, *C. micrantha* plants flower from November to December (peak flowering in November), and *C. flavida* flowers from September to November (peak flowering in September to October). This phenological difference is genetically based, as it persists for these plants when growing in a common garden of Golden Camellia Park in Nanning, Guangxi. Accordingly, the ecological novelty and distinct phenology attribute of *C.* “*ptilosperma*” most likely arose through the combination of genetic material from those two parents.

Notably, the specific traits found in *C.* “*ptilosperma*” could serve as an effective barrier to genetic exchange with its parents, contributing to its persistence, and thus promoting conditions for the establishment a new adaptive lineage. Likewise, limestone karst areas provide an essential habitat suite for the generation of hybrid plants, given that novel ecological opportunities are crucial for the origin of hybrid species [14, 17]. Karst landforms are generally covered by discontinuous and thin soils, with a high infiltration and underground drainage system, resulting in the creation of a variety of microhabitats [51]. Soil nutrients and moisture were heterogeneity along the topography of karst ecosystems [23, 46, 79, 80]. The complex karst terrain provides various vacant ecological niches for hybrids to avoid competition and/or gene flow from their parental populations.

## Conclusion

We found evidence for natural hybridization between *C. micrantha* and *C. flavida* that gave rise to hybrid population, *C.* “*ptilosperma*”. The novel hydraulic traits fixed in *C.* “*ptilosperma*” explain its distinct ecological niche, which lies outside its parental ranges. We highlight the role of hybridization in facilitating the evolution of hybrid population with unique phenological and ecological. This study reveals a case of natural hybridization that facilitated lineage diversification in yellow camellias.

## Methods

### Population sampling

A total of 10 populations of *C. flavida* (n = 91 individuals) and 6 populations of *C. micrantha* (n = 53 individuals) were sampled from their natural collection sites (hereafter referred to as populations) in southwestern Guangxi, China (Fig. 1a). The samples collected spanned the entire distribution range of either species. Unfortunately, wild populations of MZ-SJ (*C.* “*ptilosperma*”) have been severely destroyed, the samples for it used in this study could only be collected from just 17 individuals. All the studied taxa were diploid (2n = 30) [81].

### DNA extraction, library preparation and sequencing

Fresh leaf tissues were collected from all the sampled plants and preserved on dry ice for sequencing. Total genomic DNA was extracted from each tissue sample by using a modified cetyltrimethylammonium bromide (CTAB) protocol [82] and then double digested with the restriction enzymes *Eco*RI and *NIa*III. Paired-end 150 bp reads were sequenced using a NovaSeq 6000 (Illumina) platform. The raw reads obtained from each sample were deposited at the NCBI Sequence Read Archive (SRA) database (accession number: PRJNA770534).

### Bioinformatic data processing

The sequences were assembled into *de novo* loci using the STACKS v2.54  pipeline [83] for obtaining the SNP data. The *process_radtag* module of STACKS was used for quality-filtered and demultiplexing the raw fastq reads. In order to remove the tags with intact restriction enzyme cut sites and ambiguous barcode near the 3′ ends, the sequences were trimmed to a length of 135 bp using the SEQTK tool [84]. The clean reads thus obtained were further assembled to stacks (*ustacks*), which were used to build a catalog (*cstacks*) for variant calling. In these two steps, two main parameters (M parameter of *ustacks*, and n parameter of *cstacks*) were identical to those suggested previously for use in phylogenomics of yellow camellias [45], which were determined based on the protocol described by Rochette and Catchen [85]. All the samples in the population map were matched against the catalog using the *sstacks*. Pair-end reads were associated with each single-end locus using *tsv2bam* and assembled into contigs using *gstacks*. The output SNP data files were exported using the *populations* in STACKS. The loci that were sequenced from at least 80% of the individuals in each population (-r 0.8) in at least 13 populations (-p 13) were retained.

The original variants were filtered for generating a final SNP dataset using the VCFtools v0.1.16 software [86]. The following criteria were used for generating the SNP dataset: (1) SNPs with minimum minor allele frequency of 0.05, SNPs with less than 20% missing data, and only biallelic sites were retained; (2) markers significantly deviated from Hardy-Weinberg equilibrium (p < 0.05) were exclude.

### Population structure and admixture

A population genomic approach was employed for analyzing the population genetic structure in this study, and the hybrids were identified using the ADMIXTURE program [87]. The analysis was repeated 100 times, from K = 2 to K = 10. We ran a fivefold cross-validation (CV) to assessed the best fit number of clusters (based on the K value). Apart from clustering analyses with the ADMIXTURE program, principal component analysis (PCA) was performed in this study using the EIGENSOFT package of the smartpca program [88], and the results were visualized using R v3.6.3 (https://www.r-project.org/). The pattern of genetic clustering was subsequently visualized by creating population splits based on the uncorrected *P*-distance between individuals using the Neighbor-Net algorithm of SplitTree4 program [89].

### Genetic statistics

Genetic statistics, including the average values of major allele frequency (P), observed heterozygosity (*H*_*O*_), expected heterozygosity (*H*_*E*_), and nucleotide diversity (π) across loci, were estimated using STACKS v2.54 [83]. Population differentiation within and between species were measured by the pairwise genetic differentiation parameter (*F*_*ST*_), and the significance of the observed *F*_*ST*_ was determined using 10,000 permutations in the Arlequin program [90].

### Testing hybrid origins with ABC

Based on the ADMIXTURE, PCA and Neighbor-net tree results, four genetic group were defined. These consisted of *C. micrantha*, a population referred to as *C.* “*ptilosperma*” (i.e., population MZ-SJ), two intraspecific lineages of *C. flavida* that included the populations NG-LR and NG-GLA, and all remaining populations. We used approximate bayesian computation (ABC) to compare different evolutionary scenarios for the origin of population MZ-SJ and populations NG-LR and NG-GLA of *C. flavida* using the software package DIYABC v.2.1.0 [91]. A total of ten scenarios were tested (Fig. S2). Under scenarios 1, 2, 3 and 4, the lineages diverge from *C. micrantha*/*C. flavida* and hybridize with the second species. Under scenarios 5 and 6, the MZ-SJ population/*C. flavida* populations NG-LR and NG-GLA originate from crosses between *C. micrantha* and *C. flavida* then respectively follow introgression from *C. micrantha* to *C. flavida*. Under scenarios 7 and 8, the lineages arise from a single origin based on a hybridization event between *C. micrantha* and *C. flavida* (the MZ-SJ population and *C. flavida*’s NG-LR and NG-GLA populations diverge due to cladogenesis from this hybrid lineage), whereas they diverge from *C. flavida* under scenarios 9 and 10, respectively.

The variant call format (VCF) file of the unlinked SNPs was converted to DIYABC format using the Python script vcf2DIYABC.py available from https://github.com/loire/vcf2DIYABC.py. To meet the requirements of DIYABC, SNPs were removed if they include monomorphic loci. For each prior, we set the interval for population sizes and divergence time to 10–10^5^, and that for admixture rates to 0.001–0.999, because we lacked sufficient knowledge about the population sizes, divergence times, and admixture rate of *Camellia* species. A total of 25 summary statistics including genetic diversity, pairwise sample *F*_*ST*_, and Nei’s distance as well as various admixture summary statistics were used to compare the observed versus simulated data [92]. For each scenario 10^6^ simulations were performed, from which we estimated parameter posterior distributions by taking the 1% of simulated datasets closest to the observed dataset for use in local linear regression. To compare the posterior probability of the 10 scenarios, the 10 000 (1%) simulated datasets closest to the observed dataset were selected for use in logistic regression and 500 for use with the direct approach.

After choosing the best model, the posterior distribution of each parameter was also estimated by taking 1 000 (1%) simulated datasets closet to the observed dataset for the local linear regression and applying a logit transformation to a given parameter’s values. The goodness-of-fit of the tested scenarios was assessed implementing the model-checking function of DIYABC. The PCA was carried out to visually assess the position of the observed dataset vis-à-vis the simulated datasets.

### Ecophysiological traits

The study site was located at Nonggang National Natural Reserve, Pingxiang Munipality and Daqing Mountain, Longzhou county, Guangxi, Southern China (21.82° – 22.53° N, 106.74° – 107.23° E). The study region is profoundly influenced by the subtropical monsoon. The mean annual temperature is 22 °C; precipitation ranges from 1150 to l550 mm per year, interrupted by a dry season from October to April [26]. The selection of samples used for hydraulic measurements was based on the results from the ADMIXTURE program (using K = 2), and consisted of individuals from populations of *C. flavida* (NG-LT and NG-LR), *C. micrantha* (XH-BS and BY-NP), and *C.* “*ptilosperma*” (MZ-SJ). Ecophysiological trait data were obtained from three sets of measurements. The first being leaf pressure–volume (P–V) curves obtained for each taxon to determine the relation of its leaf-level water potential at the turgor loss point to physiological drought tolerance. The second was hydraulic capacitance, assumed here to be associated with water storage strategy. The third set of measurements comprised wood anatomy traits, which are often tightly linked to the water availability of plants. We measured all the hydraulic traits of plants during the dry season (in October 2021). All measurements were performed in the laboratory of Experimental Center of Tropical Forests, Chinese Academy of Forestry (Pingxiang Munipality, Guangxi, China). Materials were moved into the laboratory within an hour of sampling.

### Leaf pressure- volume (P-V) curves

The leaf P-V curves were generated using the bench drying method [93]. For each taxon, five short leaf-bearing, sun-exposed branches were selected from five mature individuals in the early morning (one per individual plant). The collected branches were sealed in black plastic bags after cut off under water in a bucket, and immediately transported to the laboratory. A segment of approximately 10 cm was removed from the cut end under water. The PMS-1505D pressure chamber (Corvallis, OR, USA) was used for determining the water potential after 2 h of rehydration. The weight and water potential of the leaves were periodically estimated during dehydration. The final dry weight of the samples was determined by oven drying at 60 °C for 48 h. The weight of the leaves (g) was measured using a balance, and the leaf area (m^2^) was measured using a scanner.

### Measurement of capacitance

The capacitance was measured according to the methods described by Jupa, Plavcová, Gloser and Jansen [94]. Five old branches or roots, approximately 1 cm in diameter and 15 cm in length, were collected from five mature and healthy individuals per taxon. Freshly excised segments of branches and roots were wrapped in a plastic bag and immediately transported to the laboratory for further analyses. After removing the bark, the cambium and pith were separated from the segments, and the remaining tissues were cut into 2-cm-long segments and vacuum- infiltrated with distilled water for 12 h. After rehydration, the remaining segments were shortened to a 5-mm length to quantify water potential with a WP4C water potential meter (Meter, Hopkinsville, USA); this apparatus was calibrated prior to measurements on a daily basis. The weight of the segments and their water potential were periodically measured during dehydration stage until the water potential fell below − 8 MPa. Next, the volume of each sample was determined using the water displacement method, after which all samples were oven-dried at 70 °C for at least 48 h for their dry mass determination. Relative water content (RWC) was calculated using this equation:

$$\text{R}\text{W}\text{C}=\frac{Wf - Wd}{Ws - Wd}$$

where *W*_*f*_ and *W*_*d*_ denote the weight of a given sample before and after its dehydration, respectively, and *W*_*s*_ is the weight of the sample when fully saturated. Cumulative water release (CWR, kg m^− 3^) was calculated as follows:

$$\text{C}\text{W}\text{R}=\left(1-\text{R}\text{W}\text{C}\right)\times \left(\text{W}\text{s}-\text{W}\text{d}\right)\times \left(\frac{{\rho }\times 1000}{\text{W}\text{d}}\right)$$

where ${\rho }$ is the density of the wood (g/cm^− 3^), this expressed as the ratio of dry mass of a sample to its volume. To describe the relationship between the CWR and water potential (Ψ) of each *Camellia* species, the data were fitted to the hyperbolic function:

$$\text{y}=\frac{ax}{b+x}$$

where *a* is the best-fitting parameter denoting the value of CWR in the asymptotic region of the curve, and *b* is the best-fitting parameter for the values of Ψ. The shape of the curve was divided into two phases according to the *b* parameter value: phase I (from Ψ = 0 to *b* MPa) and phase II (from Ψ = *b* to − 8 MPa). Phase I was characterized by the initial rapid release of water, concurrent with a decline in Ψ, and phase II corresponded to the period of gradual water release. Finally, the capacitance in the two distinct phases was calculated as the ratio of ∆CWR to ∆Ψ:

$$\text{C}=\frac{{\Delta }\text{C}\text{W}\text{R}}{{\Delta }{\Psi }}$$

### Wood anatomy

**Fig. 5:**
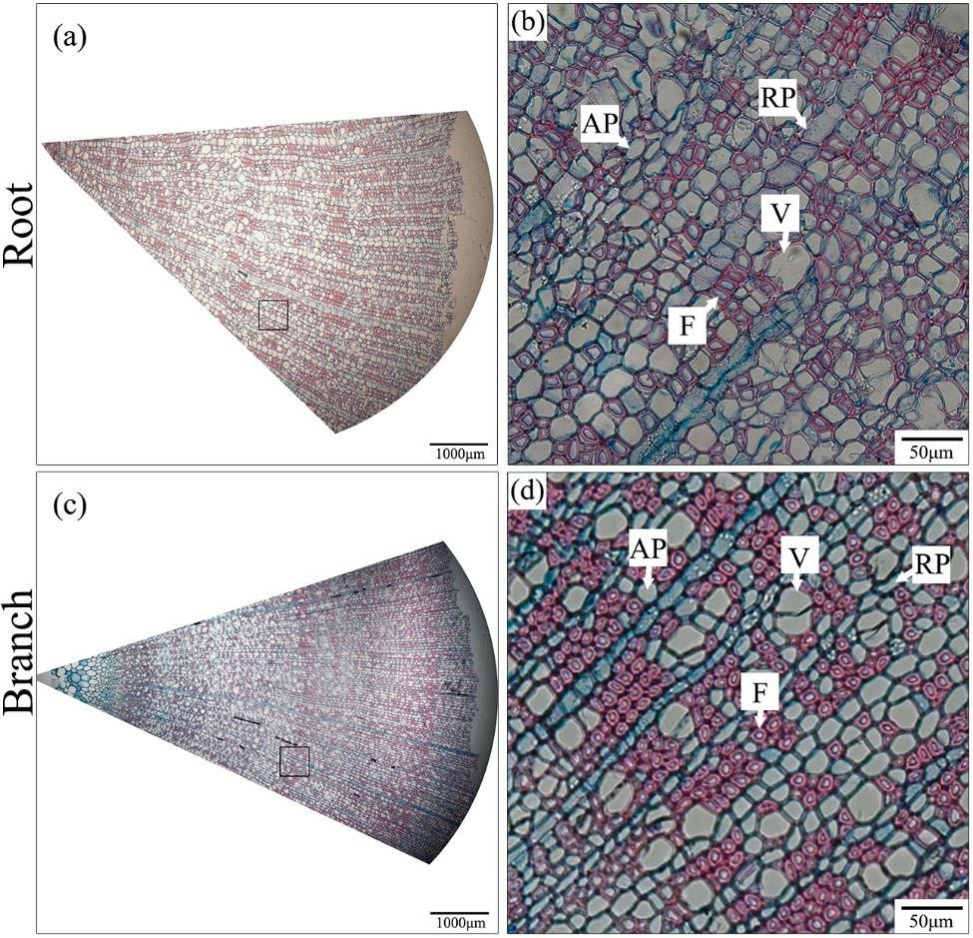
Representative light microscopy images of transverse sections of wood anatomy of the yellow camellias studied herein. Axial parenchyma (AP), ray parenchyma (RP), vessel (V), fiber (F)

After measuring the hydraulic capacitance, the remaining fresh branches and roots were used to analyze the anatomical characteristics of each sample’s woody tissue. To do this, transverse sections (approximately 25 μm) were prepared using a sliding microtome (SM2010R, Leica, Wetzlar, Germany). under a light microscope (Nikon Eclipse 50i, Nikon, Tokyo, Japan) and the obtained images then analyzed in ImageJ software [95]. vessel diameter (D) and relative proportion of various cell types in the wood, namely, vessels (V), fibers (including tracheids) (F), and ray and axial parenchyma (RP and AP), were determined from more than 10 fields per transverse section. In Fig. 5 are representative images of wood anatomy obtained by light microscopy.

## Electronic supplementary material

Below is the link to the electronic supplementary material.

Additional file 1

Additional file 2

## Data Availability

statement. The datasets generated or analysed during the current study are available in the NCBI Sequence Read Archive and can be found under BioProject PRJNA770534 with accession nos. SAMN30101064–SAMN30101208, SAMN22253095–SAMN22253102, SAMN22253085–SAMN22253086, SAMN22253119–SAMN22253120, SAMN22253123–SAMN22253126 (https://www.ncbi.nlm.nih.gov/bioproject/PRJNA770534).

## Abbreviations

ABC: approximate bayesian computation
AP: axial parenchyma
CTAB: cetyltrimethylammonium bromide
CV: cross-validation
CI: confidence interval
D: diameter
ddRAD: double digest restriction-site associated DNA
*F*_*ST*_: genetic differentiation index
F: fibers
*H*_*O*_: observed heterozygosity
*H*_*E*_: expected heterozygosity
π: nucleotide diversity
P: major allele frequency
PCA: principal component analysis
P–V: pressure–volume
RP: ray parenchyma
SNP: single nucleotide polymorphism
SD: standard deviation
SRA: Sequence Read Archive
VCF: variant call format
V: vessels
WGD: whole genome duplication
RWC: relative water content
Ψ: water potential
Ψ_TLP_: the water potential at turgor loss point
