# Supplementary material for: Genetic and ecophysiological evidence that hybridization facilitated lineage diversification in yellow Camellia (Theaceae) species: a case study of natural hybridization between C. micrantha and C. flavida
Source: BMC Plant Biol. 2023 Mar 22;23:154. doi: 10.1186/s12870-023-04164-4 (PMC10031943; doi:10.1186/s12870-023-04164-4)
Supplement: Supplementary file 1 — Additional file 1 [file 12870_2023_4164_MOESM1_ESM.pdf]

**Additional file 1:**

Table S1. Geographical location (latitude and longitude), sample size, and genetic statistics (*P*, $H_O$, $H_E$, and π) of the populations of *C. micrantha, C.* "*ptilosperma*" and *C. flavida*; n, number of individuals; the average values of *P*, $H_O$, $H_E$, and π across loci are listed.

Table S2. Water content in the branches and roots of the studied yellow camellias. b, parameters value from the best-fitting hyperbolic equation y = *a*x/(*b* + x) based on the cumulative water release curves plotted in Fig. S5. $C_I$ and $C_{II}$, capacitance in phases I and II (kg $m^{-3}$ $MPa^{-1}$); $WC_I$, amount of water released during phase I (from Ψ = 0 to *b* MPa) of the moisture release curve (kg $m^{-3}$). B, branch; R, root.

Table S3. Anatomical parameters of branches and roots of the studied yellow camellias. D, vessel diameter (μm); V, fraction of vessels; F, fraction of fibres/tracheids; AP, fraction of axial parenchyma; RP, fraction of ray parenchyma. Values are the mean ± SD.

Table S1. Geographical location (latitude and longitude), sample size, and genetic statistics (*P*, $H_O$, $H_E$, and π) of the populations of *C. micrantha, C.* "*ptilosperma*" and *C. flavida*; n, number of individuals; the average values of *P*, $H_O$, $H_E$, and π across loci are listed.

| Taxa | Population Code | n | Latitude | Longitude | Variant positions | | | |
|---|---|---|---|---|---|---|---|---|
| | | | | | *P* | $H_O$ | $H_E$ | π |
| *C. micrantha* | BY-NP | 11 | 22.17 | 106.79 | 0.96926 | 0.04936 | 0.04418 | 0.04663 |
| *C. micrantha* | BY-PS | 9 | 22.25 | 106.74 | 0.97131 | 0.04978 | 0.03687 | 0.03911 |
| *C. micrantha* | XH-BS | 9 | 22.16 | 106.88 | 0.96993 | 0.05113 | 0.04257 | 0.04523 |
| *C. micrantha* | XH-FL | 7 | 22.15 | 106.87 | 0.97491 | 0.04132 | 0.03396 | 0.03729 |
| *C. micrantha* | BY-PT | 8 | 21.84 | 107.15 | 0.97282 | 0.04579 | 0.03633 | 0.03904 |
| *C. micrantha* | BY-WH | 9 | 21.82 | 107.23 | 0.98072 | 0.03473 | 0.02744 | 0.02923 |
| *C.* "*ptilosperma*" | MZ-SJ | 17 | 22.12 | 106.87 | 0.95588 | 0.06344 | 0.06901 | 0.0715 |
| *C. flavida* | NG-LR | 9 | 22.25 | 107.08 | 0.9282 | 0.11475 | 0.10351 | 0.11002 |
| *C. flavida* | NG-GLA | 8 | 22.28 | 107.10 | 0.93677 | 0.10418 | 0.09133 | 0.09815 |
| *C. flavida* | NG-LLS | 8 | 22.42 | 107.03 | 0.88498 | 0.19531 | 0.17003 | 0.18196 |
| *C. flavida* | NG-LT | 9 | 22.42 | 107.00 | 0.88345 | 0.1959 | 0.16705 | 0.1774 |
| *C. flavida* | DY-NZ | 10 | 22.43 | 107.01 | 0.8747 | 0.21054 | 0.17911 | 0.18926 |
| *C. flavida* | NG-MQ | 10 | 22.43 | 106.97 | 0.87828 | 0.2069 | 0.17912 | 0.18901 |
| *C. flavida* | NG-ND | 9 | 22.46 | 106.94 | 0.87824 | 0.20584 | 0.17091 | 0.18135 |
| *C. flavida* | NG-SC | 9 | 22.52 | 106.87 | 0.89179 | 0.17844 | 0.15567 | 0.16565 |
| *C. flavida* | NG-LU | 10 | 22.52 | 106.85 | 0.88688 | 0.1909 | 0.16304 | 0.17235 |
| *C. flavida* | NG-LD | 9 | 22.53 | 106.82 | 0.88989 | 0.18083 | 0.15741 | 0.16721 |

Table S2. Water content in the branches and roots of the studied yellow camellias. b, parameters value from the best-fitting hyperbolic equation y = *a*x/(*b* + x) based on the cumulative water release curves plotted in Fig. S5. $C_I$ and $C_{II}$, capacitance in phases I and II (kg $m^{-3}$ $MPa^{-1}$); $WC_I$, amount of water released during phase I (from $\Psi = 0$ to *b* MPa) of the moisture release curve (kg $m^{-3}$). B, branch; R, root.

| Taxa | Population | Organ | b | $C_I$ | $C_{II}$ | $WC_I$ |
|---|---|---|---|---|---|---|
| *C. micrantha* | XH-BS | B | -0.72 | 379.69 | 22.10 | 130.61 |
| | | R | -2.42 | 128.65 | 39.40 | 208.33 |
| | BY-NP | B | -0.61 | 166.64 | 25.82 | 108.70 |
| | | R | -1.81 | 64.79 | 31.85 | 135.14 |
| *C.* “*ptilosperma*” | MZ-SJ | B | -2.81 | 87.60 | 22.60 | 157.25 |
| | | R | -2.32 | 86.35 | 17.37 | 193.72 |
| *C. flavida* | NG-LT | B | -2.99 | 80.23 | 23.43 | 196.17 |
| | | R | -1.37 | 177.15 | 28.37 | 121.95 |
| | NG-LR | B | -2.49 | 52.71 | 33.78 | 157.93 |
| | | R | -1.85 | 131.94 | 25.56 | 246.52 |

Table S3. Anatomical parameters of branches and roots of the studied yellow camellias. D, vessel diameter (μm); V, fraction of vessels; F, fraction of fibres/tracheids; AP, fraction of axial parenchyma; RP, fraction of ray parenchyma. Values are the mean ± SD.

| Taxa | Population | Organ | D (μm) | V (%) | F (%) | AP (%) | RP (%) |
|---|---|---|---|---|---|---|---|
| *C. micrantha* | XH-BS | B | 24.54 ± 2.13 | 9.25±2.05 | 41.75±6.08 | 20.52±6.83 | 27.85±9.82 |
| | | R | 20.42 ± 3.52 | 11.69±2.85 | 34.20±6.24 | 20.26±2.97 | 33.85±5.83 |
| | BY-NP | B | 24.04 ±2.33 | 8.42±2.37 | 43.91±5.88 | 19.26±5.81 | 28.42±8.41 |
| | | R | 31.38 ± 4.35 | 6.73±6.80 | 43.00±15.42 | 19.93±13.01 | 31.79±10.53 |
| *C.* “*ptilosperma*” | MZ-SJ | B | 18.07 ± 2.27 | 6.53±2.89 | 41.70±3.43 | 20.99±2.37 | 30.78±2.48 |
| | | R | 22.22 ± 1.68 | 8.14±1.01 | 33.97±7.20 | 13.77±2.98 | 44.09±8.73 |
| *C. flavida* | NG-LT | B | 19.20 ± 1.09 | 6.71±3.27 | 38.84±8.16 | 21.37±2.30 | 33.08±8.99 |
| | | R | 27.22 ± 1.42 | 7.92±3.18 | 30.11±4.52 | 23.41±4.33 | 38.57±9.66 |
| | NG-LR | B | 21.68 ± 1.78 | 8.32±3.26 | 39.58±3.44 | 23.02±4.69 | 29.07±8.03 |
| | | R | 28.16 ± 5.22 | 9.15±3.20 | 39.56±4.98 | 17.74±2.89 | 33.56±3.26 |
