# Supplementary material for: Genetic and ecophysiological evidence that hybridization facilitated lineage diversification in yellow Camellia (Theaceae) species: a case study of natural hybridization between C. micrantha and C. flavida
Source: BMC Plant Biol. 2023 Mar 22;23:154. doi: 10.1186/s12870-023-04164-4 (PMC10031943; doi:10.1186/s12870-023-04164-4)
Supplement: Supplementary file 2 — Additional file 2 [file 12870_2023_4164_MOESM2_ESM.pdf]

Additional file 2:

Fig. S1. Cross-Validation (CV) error plot indicating the choice of the appropriate K value.

Fig. S2. Ten scenarios tested in the DIYABC analysis based on the SNPs data. The current population sizes of *C. micrantha, C.* "*ptilosperma*", and two groups of *C. flavida* were denoted as N1, N2, N3 and N4, respectively. N5, the population size of the lineage diverges from *C. micrantha*/*C. flavida*. Na, the population size of the ancestral lineage. t1–t4, the divergence times for the depicted event. r1 and r2 refer to the rate of admixture.

Fig. S3. The prior and posterior distributions for each parameter obtained using DIYABC. Along the y-axis are the probability densities of the priors and posteriors.

Fig. S4. Principal Component Analysis (PCA) obtained by DIYABC. Small empty circles represent datasets simulated from priors, large filled circles represent datasets simulated from posteriors and the large yellow circle represents the observed dataset

Fig. S5. The values of capacitance in the two phases of the water release curves of the studied yellow camellias. Panels (a) and (b) represent the water release curves of the branches and roots, respectively. B, branch; R, root. Ψ, water potential (MPa); CWR, cumulative water release (kg m^{-3}). Vertical lines were the b parameters from hyperbolic functions, which mean the dividing line between Phase I and Phase II. The population codes are described in Table S1.

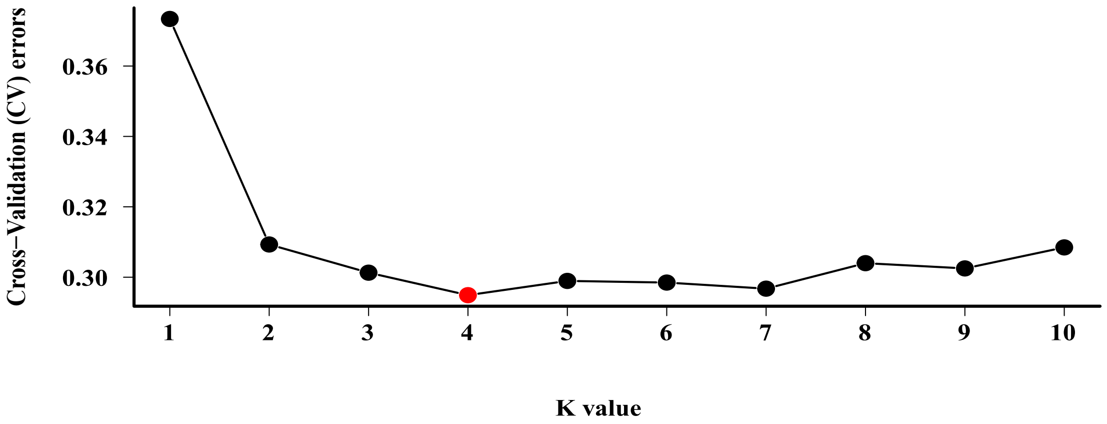

Fig. S1. Cross-Validation (CV) error plot indicating the choice of the appropriate K value.

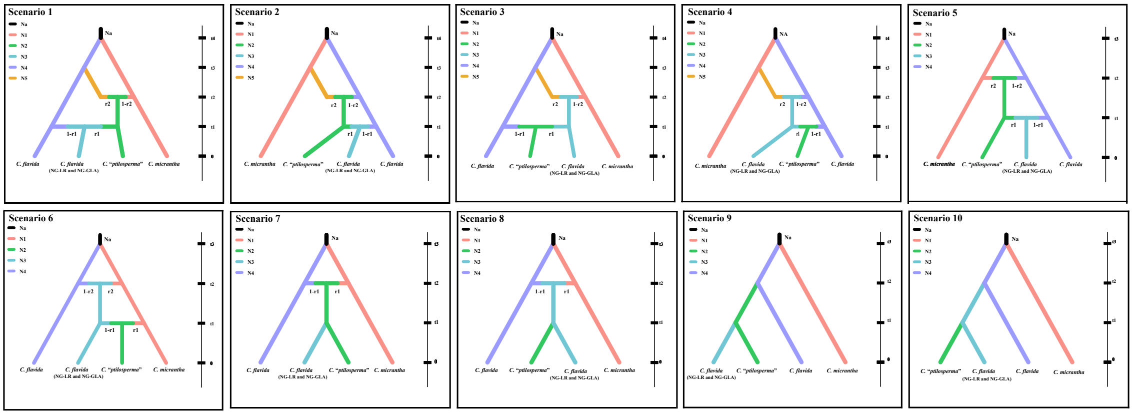

Fig. S2. Ten scenarios tested in the DIYABC analysis based on the SNPs data. The current population sizes of *C. micrantha, C.* "*ptilosperma*", and two groups of *C. flavida* were denoted as N1, N2, N3 and N4, respectively. N5, the population size of the lineage diverges from *C. micrantha*/*C. flavida*. Na, the population size of the ancestral lineage. t1–t4, the divergence times for the depicted event. r1 and r2 refer to the rate of admixture.

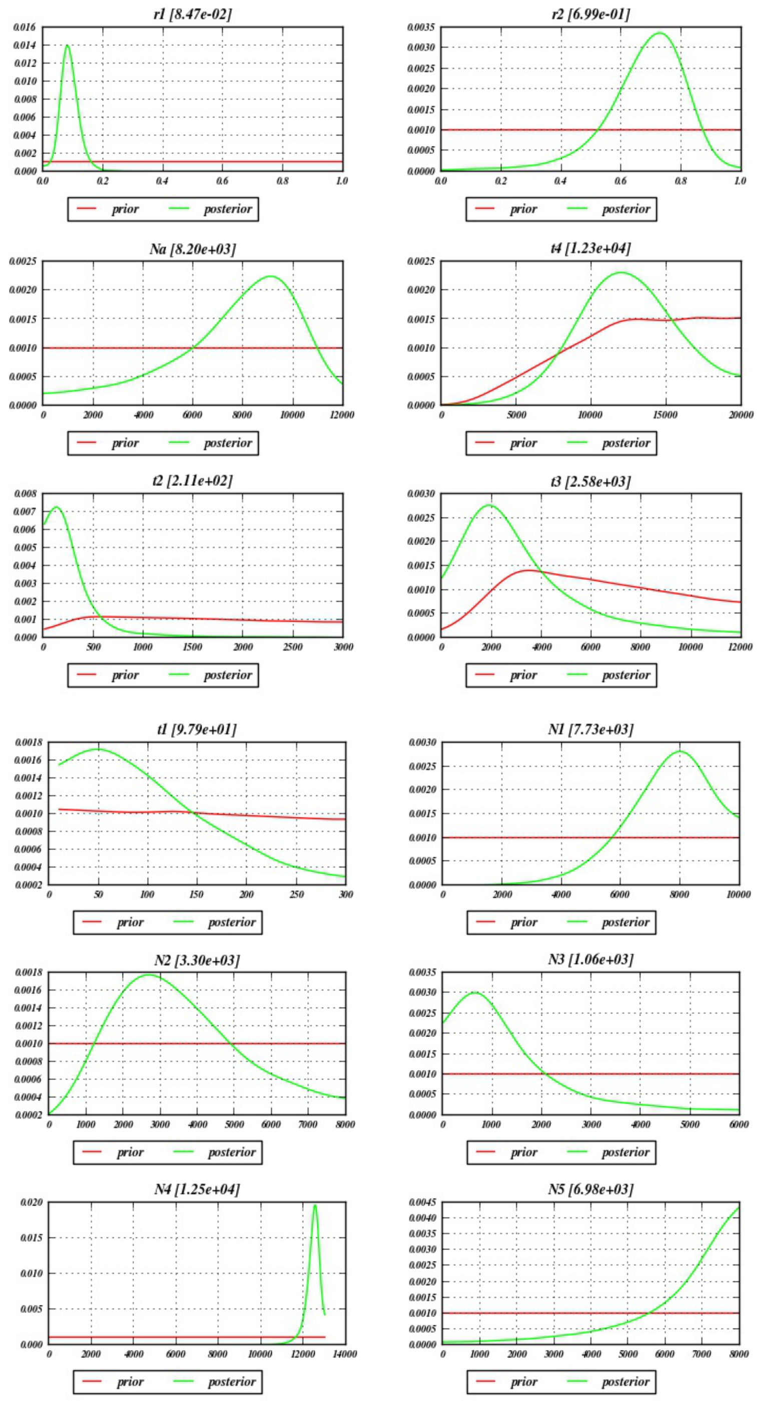

Fig. S3. The prior and posterior distributions for each parameter obtained using DIYABC. Along the y-axis are the probability densities of the priors and posteriors.

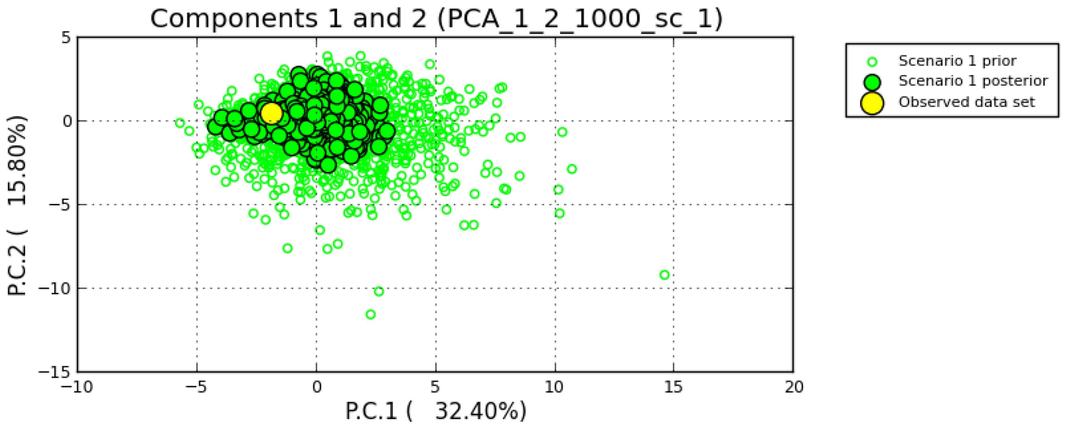

Fig. S4. Principal Component Analysis (PCA) obtained by DIYABC. Small empty circles represent datasets simulated from priors, large filled circles represent datasets simulated from posteriors and the large yellow circle represents the observed dataset.

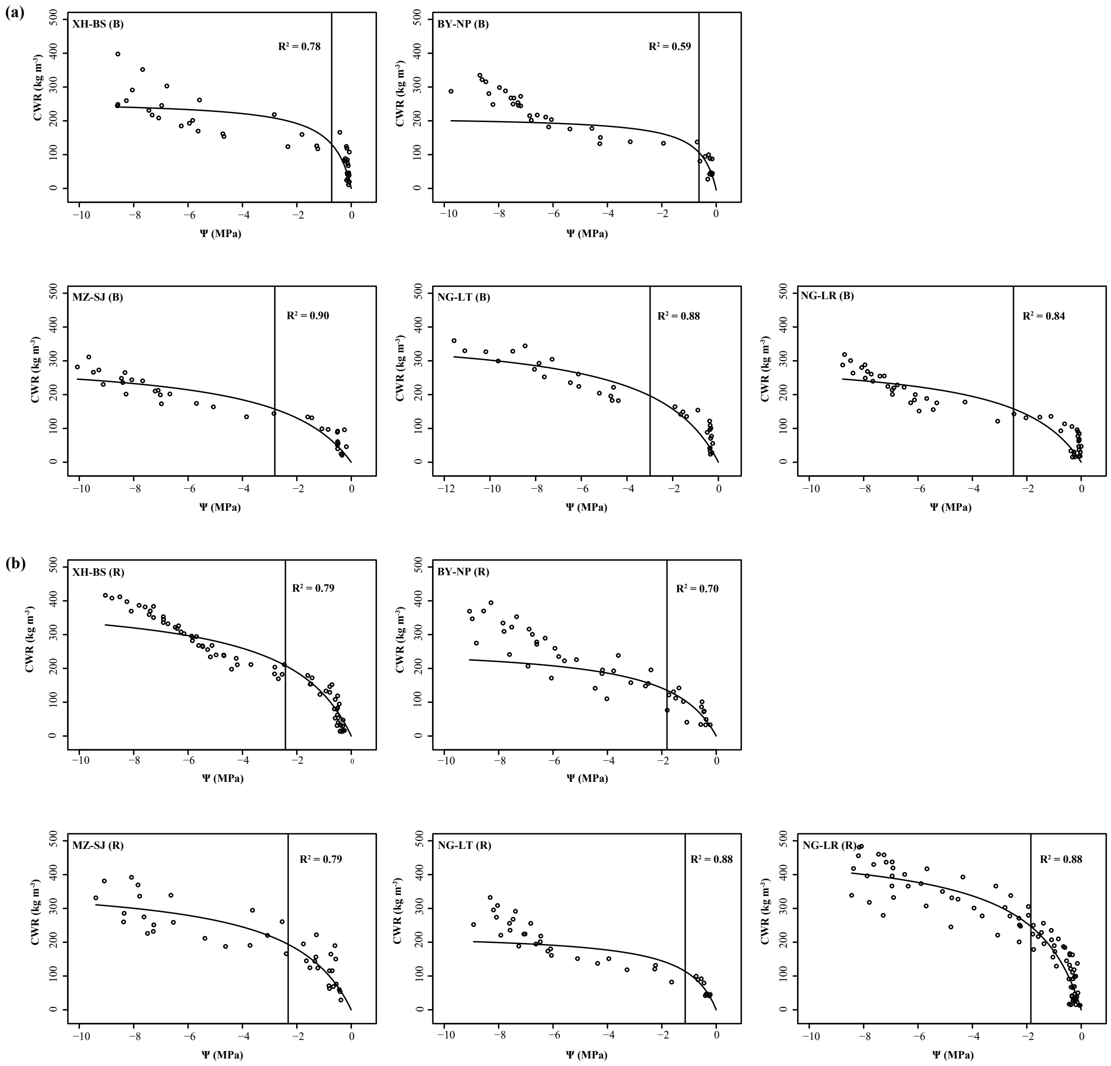

Fig. S5. The values of capacitance in the two phases of the water release curves of the studied yellow camellias. Panels (a) and (b) represent the water release curves of the branches and roots, respectively. B, branch; R, root. Ψ, water potential (MPa); CWR, cumulative water release (kg m^{-3}). Vertical lines were the b parameters from hyperbolic functions, which mean the dividing line between Phase I and Phase II. The population codes are described in Table S1.
